# Trans-scleral cryotherapy in the management of retinal hemangioblastomas

**DOI:** 10.1007/s00417-026-07208-1

**Published:** 2026-04-11

**Authors:** Geoffrey K. Broadhead, Henry E. Wiley, Elvira Agrón, David Dao, Emily Y. Chew, Hanna Coleman, Alisa T. Thavikulwat

**Affiliations:** 1 Division of Epidemiology & Clinical Applications, National Eye Institute, National Institutes of Health, Bethesda, MD 20892, USA; 2 Save Sight Institute, The University of Sydney, Sydney, NSW, Australia; 3 Canberra Eye Surgeons, Canberra, ACT, Australia; 4 Genentech Incorporated, South San Francisco, CA, USA; 5 Columbia University, New York, NY, USA

**Keywords:** Retina, von Hippel Lindau, Retinal hemangioblastoma, Cryotherapy

## Abstract

**Purpose:**

To evaluate the efficacy of trans-scleral retinal cryotherapy in the management of retinal hemangioblastoma (RH) including in von Hippel Lindau disease (VHL).

**Methods:**

Retrospective cohort study of 24 patients (21 with VHL) receiving trans-scleral cryotherapy performed to RH lesions, with lesion characteristics and visual acuity (VA) assessed pre and post treatment using color photographs, optical coherence tomography and recorded clinical notes.

**Results:**

Thirty-six lesions in 26 eyes received cryotherapy, with a mean follow up of 6.7 ± 3.5 years. Mean pretreatment VA was 69.7 ± 20.4 letters (Snellen equivalent of 20/40); 8 (22%) lesions were classified as clusters containing multiple RHs. Twenty-one (64%) lesions underwent retreatment using any modality and 15 (57.7%) eyes had treatment success (14 total success and 1 partial success). The average VA change at 1 year for patients with ≥ 1 year of follow-up was −4.6 letters (*p* = 0.36 for VA change from baseline). In a logistic regression model, lower patient age at the time of cryotherapy trended towards increased odds of success (Odds ratio 1.085, 95% CI 1.00–1.180), but no other analyzed factor was statistically significantly associated with treatment success.

**Conclusions:**

Trans-scleral retinal cryotherapy can be effective in treating RHs. A significant proportion of these eyes progress despite cryoablative treatment.

## Introduction

Retinal hemangioblastomas (RHs) are rare, vascular tumors that arise in the retina and are often seen in association with von Hippel-Lindau disease (VHL) [1]. VHL is a rare, autosomal dominant condition that affects a range of organs, including the eye [1]. RHs represent one of the most common manifestations of VHL and may appear as single or multiple lesions at any age [2]. These lesions can grow and produce exudation, hemorrhage, and epiretinal membranes, resulting in a number of consequences, including vitreous hemorrhage, retinal detachment, and loss of vision. The risk of these consequences increases with greater RH size [2, 3].

Ablative treatment of smaller RHs is aimed at preventing RH growth and thus reducing the risk of vision-threatening complications. Laser photocoagulation is the mainstay of therapy for small peripheral lesions [3, 4]. This therapy is highly effective for peripheral lesions < 1 disc diameter (DD) and results in tumor regression, frequently after a single treatment session, without significant adverse effects. Larger RHs, however, are more difficult to manage, and are frequently not destroyed even after multiple laser sessions [3]. A number of ablative and nonablative therapies have been utilized, including photodynamic therapy (PDT), intravitreal anti-vascular endothelial growth factor (anti-VEGF) injections, surgical excision, radiotherapy, and trans-scleral cryotherapy, or combinations of these approaches [3-9].

Varying degrees of success have been reported in case reports or small series with each of these therapies. PDT and anti-VEGF injections may result in partial regression and/or a decrease in exudation, but do not usually result in complete regression [5, 6]. Cryotherapy offers the potential for complete destruction of lesions that cannot be destroyed by laser photocoagulation, but carries higher risks than laser treatment of post-treatment complications that can include exudation, hemorrhage, vitreous contraction, and epiretinal membrane formation [10]. Successful surgical excision has been described in case reports and series, but carries risks of hemorrhage, retinal detachment and scarring [3, 7, 8]. Similarly, radiotherapy has been reported to shrink lesion size, but with variable visual outcomes [11, 12].

Trans-scleral cryotherapy has some advantages over radiotherapy or surgery, including an ability to be performed in both inpatient and outpatient settings, and the potential to treat larger lesions without the need for intraocular surgery or radiotherapy and the associated risks of those therapies. However, published data about the long-term outcomes and factors influencing success or failure of cryotherapy for RH are limited. This retrospective study evaluated the long-term efficacy of cryotherapy as a treatment for RHs, and as a means of preventing significant vision-threatening complications.

## Methods

Clinical records from the National Eye Institute (NEI) clinic at the National Institutes of Health (NIH) in Bethesda, MD were searched for all participants with a diagnosis of VHL or RH between January 2004 and July 2021. Inclusion criteria included: diagnosis of RH receiving cryotherapy on at least one occasion at the NIH and follow up of at least 1 month following cryotherapy treatment. Exclusion criteria were prior cryotherapy to the same lesion at another center within 6 months of baseline NIH visit or follow-up less than 1 month after treatment. Lesions refractory to previous other types of treatments including laser were included. This study preceded belzutifan availability and no patient received belzutifan during the study period.

### Trans-scleral cryotherapy treatment

Treatments were performed to one or more lesions in each eye and were performed in either the clinic or operating room setting. A double freeze-thaw technique using visualization by indirect ophthalmoscopy was employed, with an objective for each application of causing complete whitening of the entirety of each discrete RH within the field of treatment, with a variable amount of ice formation in the vitreous cavity overlying the lesion. Relevant factors for choice of the operating room setting included lesion location (with post-equatorial lesions managed using limited conjunctival peritomy to provide access and proper placement of the probe tip); patient age (with pediatric patients treated in the operating room); and patient preference based on comfort (with monitored anesthesia care and retrobulbar injection used for patients not appropriate for clinic treatment). Local anesthesia typically consisted of subconjunctival injection for peripheral lesions treated in clinic, and retrobulbar injection for lesions treated in the operating room. Systemic oral or intravenous corticosteroids were employed in an effort to mitigate expected postsurgical exudation/exudative retinal detachment. Corticosteroids were typically initiated on the day of treatment just before the procedure and were usually continued for 3–7 days at a tapering dose. Periocular corticosteroids were generally not used except in circumstances where a prolonged effect was needed for factors in addition to control of early postsurgical exudation. Consideration was given to positioning the patient overnight after treatment to minimize the risk of dependent migration of postsurgical exudation into the macula. All patients were seen for follow-up on post-operative day 1.

### Data collection

At each visit, patients underwent an ophthalmic examination including measurement of visual acuity (VA), intraocular pressure (IOP), and dilated retinal examination. Imaging with spectral domain optical coherence tomography (OCT), color fundus photography (CP), ultrawidefield color imaging (UW-CP), and fluorescein angiography was performed as clinically indicated.

Demographic data on age, gender, clinical VHL status and prior therapy to the same or other RHs in either eye were collected at baseline. For each recorded follow-up visit, clinical data were collected using a combination of clinical records data and imaging findings as appropriate and as recorded in the NEI database. Incidence of certain treatment-related or RH-related adverse events, specifically retreatment with cryotherapy, other ocular surgery, retinal detachment or fundus-obscuring vitreous hemorrhage, were also collected. Where data were unavailable at a specific time point, they were considered missing for the purposes of analysis.

### Grading

**Fig. 1:**
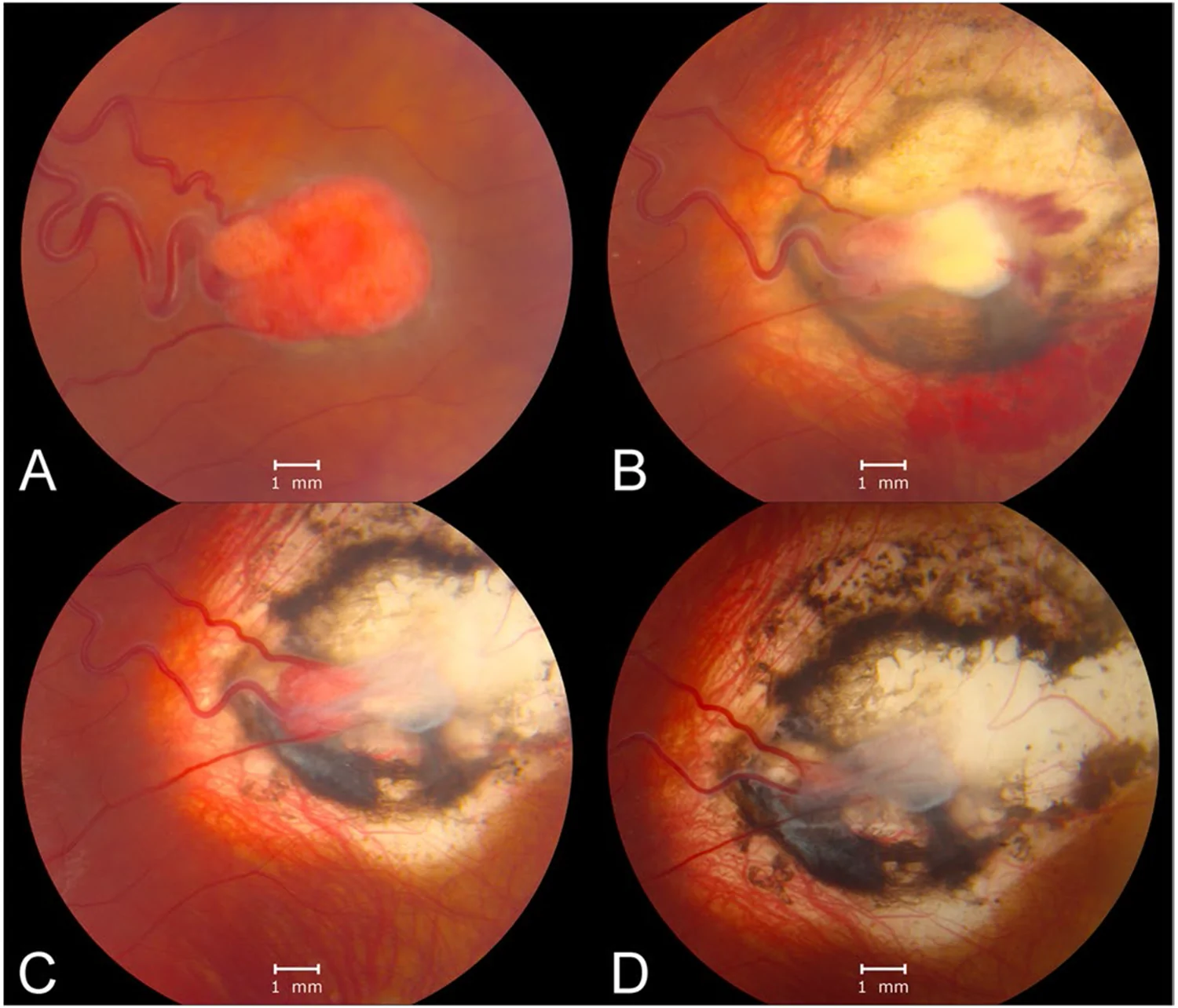
Color fundus photography of the clinical course of a lesion that was classified as successfully treated with trans-scleral cryoablation. (**A**) Single retinal hemangioblastoma (A) measuring ≥ 3 disc diameters (≥ 4.5 mm) in size at baseline with severe dilation and tortuosity of feeding and draining vessels, no lipid exudate, mild SRF, and no vitreous traction or pre-retinal fibrosis. (**B**) Four months following cryotherapy, there is early regression of the RH with improvement of vessel dilation and tortuosity to a moderate degree, but persistence of a vestige of the RH at the base of the inactive main lesion. The patient underwent intravitreal ranibizumab injections for subretinal neovascularization and macular oedema. (**C**) At 1 year following cryotherapy, the RH vestigial nodule at the base of the lesion appears more active and the decision was made to proceed with laser ablation. (**D**) One year following laser ablation, the lesion appeared inactive.

**Fig. 2:**
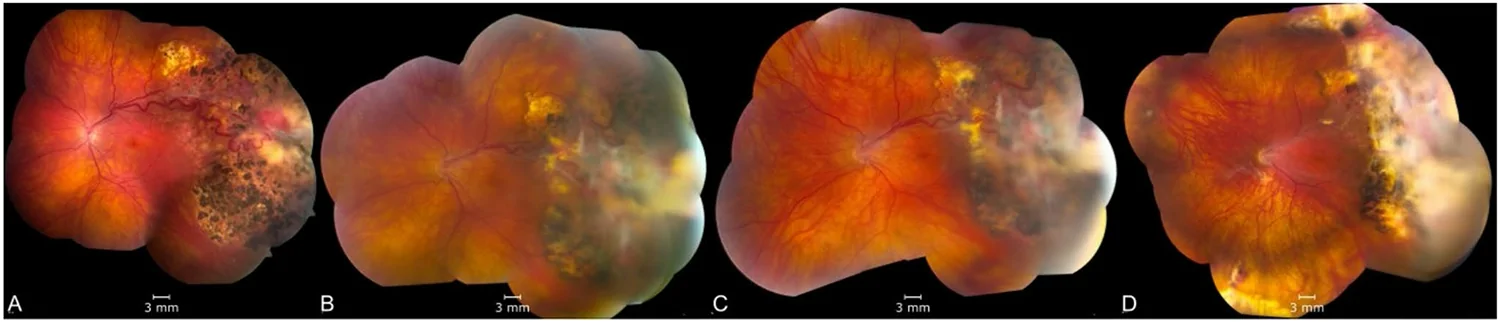
Color fundus photography of the clinical course of a lesion that was classified as not successfully treated with trans-scleral cryoablation. (**A**) Initial presentation to our clinic of a cluster lesion that had previously been treated with cryotherapy and laser ablation. (**B**) Two years after initial presentation, the lesion had progressed with peripheral retinal detachment and patient had undergone an additional session of limited cryotherapy and laser. The lesion measured 2–3 disc diameters (3.0–4.5 mm) in size with moderate feeding/draining vessel dilation and tortuosity, moderate lipid exudation, severe subretinal fluid, severe preretinal fibrosis obscuring visualization of the tumor body, and mild vitreous traction. (**C**) Four months following cryotherapy at our institute, there was persistent peripheral retinal detachment associated with retinal traction and exudation. The patient subsequently underwent episcleral plaque radiation, followed by pars plana vitrectomy with silicone oil placement, which was later removed, and scleral buckling. (**D**) Two years following trans-scleral cryotherapy at our institute, the patient was continuing to receive intravitreal bevacizumab injections for residual subretinal fluid, but the RH appeared quiescent.

Imaging and clinical characteristics were collected at each visit and graded using pre-specified standardized criteria (see Figs. 1 and 2 as examples of lesions at various stages of treatment). Data were collected on the following factors: lesion type, lesion size, lesion location, degree of lipid exudate, degree of subretinal fluid (SRF), presence of overlying vitreous traction, presence of overlying fibrosis, degree of feeding/draining vessel dilation, presence and degree of vitreous hemorrhage, presence of retinal detachment, presence of epiretinal membrane, presence of macula exudation and presence of other RHs in the eye.

Treatment success was graded upon review of notes and available images, and success was adjudicated at the lesion level. To allow for variable follow-up, treatment success based on structural features was assessed at 3–12 months following cryotherapy. Complete success was defined as decrease in lesion size, resolution of exudation, decrease in feeding vessel caliber and tortuosity, and flattening with whitening or pigmentation of the surface to a degree indicating no signs of residual viability. Partial success was defined as an improvement in lesion activity based on the parameters above such that further treatment was predicted to have a high chance of completely inactivating the lesion. The definition for partial success allowed for cryotherapy to be considered successful if it debulked the tumor sufficiently for further laser and/or cryotherapy to achieve complete ablation; lesions requiring further non-surgical therapy (i.e. not requiring further cryotherapy or requiring retinal surgery) within the initial 3–12 months were considered to be a partial success. If the only follow-up visit after the peri-operative period was beyond 12 months, the treatment was considered successful if the lesion was completely inactive.

### Analysis

Treatment success rate was analyzed as the primary study outcome. Secondary analyses included VA and VA change from baseline at 12 months and change in clinical and imaging parameters at the same timepoint. Regression analysis was conducted to investigate the effects of age, history of prior cryotherapy, and lesion characteristics (single vs. cluster lesion type, size, location, degree of lipid exudate, degree of subretinal fluid (SRF), presence of overlying vitreous traction, presence of overlying fibrosis, and degree of feeding/draining vessel dilation) on treatment success. Procedural characteristics including the route of use of post-operative corticosteroid therapy and location of cryotherapy in an operating theatre or in clinic were also analyzed. The likelihood ratio test was used to select the final multivariate model that best fit the data [13]. Analysis was conducted using SAS software version 9.4 (SAS Institute, Inc, Cary, NC). A p-value of < 0.05 was considered statistically significant. Certain categories, notably no/mild/moderate vascular dilation, no/mild SRF, and superonasal/inferonasal lesion location, were grouped together to increase power in statistical analysis; for some analyses partial and complete success categories were also grouped together to increase power in statistical analysis.

## Results

Thirty-six lesions in 26 eyes of 24 patients met all inclusion and exclusion criteria, and demographic and baseline clinical data are shown in Table 1. Briefly, 8 (33.3%) patients were female with a mean age at treatment of 33.8 ± 12.1 years, and 28 (77.8%) lesions were present as single RHs. Average follow-up time was 6.7 ± 3.5 years with a median follow up of 8.5 years.

### Examination & imaging characteristics

Baseline examination and imaging findings are detailed in Table 1 and change in these characteristics at 1 year in 21 eyes is outlined in Table 2. Briefly, moderate or severe lipid exudate was seen in 15/36 (42%) of lesions at baseline, and a reduction in lipid at any level was noted in 38% of lesions with 1 year of follow up (21 lesions); severe vascular dilation was seen in 26/36 (72%) of lesions at baseline and a reduction in vascular dilation at any stage was observed in 53% of lesions at 1 year; and moderate to severe subretinal exudation/SRF was seen in 9/36 (25%) of eyes at baseline, with a reduction in SRF of any stage (including mild SRF) noted in 36% of eyes at 1 year.

## Treatment success/retreatment/adverse events

Complete treatment success occurred in 17 lesions (85% of successfully treated lesions; 52% of all lesions). A further 3 lesions (9% of all lesions) had partial treatment success within the first year.

Twenty-one (63.6%) lesions required additional treatment with either laser, cryotherapy, intravitreal injection of anti-VEGF agent, vitreoretinal surgery, radiotherapy or a combination of these modalities at any time including after 1 year. Of 25 lesions in eyes without a history of cryotherapy, 12 eyes had complete success and 2 had partial success. In a logistic regression model, patient age at the time of cryotherapy approached significance for odds of success (OR 1.085, 95% CI 1.00–1.180; *p* = 0.003 Table 3, **95**% CI includes 1.00 due to rounding). No other factor was associated with treatment success. There were 7 (27%) eyes that experienced a retinal detachment (6 tractional detachments including 2 combined tractional/rhegmatogenous detachments), and 5 of these events occurred within the first year after cryotherapy, with 2 retinal detachments occurring more than 1 year after treatment.

### Visual acuity

Mean baseline VA was 69.7 ± 20.4 letters (20/40 Snellen equivalent) for all eyes. Mean change in VA at 1 year was −4.6 ± 14.5 letters for those 21 eyes with 1 year of follow-up (*p* = 0.36 for change in VA at 1 year; Table 2). Mean baseline VA for patients with complete treatment success was not statistically significantly different than for those without success (77.0 letters (20/30 Snellen equivalent) vs. 59.4 (20/63 Snellen equivalent) letters, *p* = 0.27). For eyes with lesions that required retreatment, mean VA change at 1 year was −8.7 ± 18.7 letters, compared to −0.8 ± 8.5 letters for eyes with lesions that did not require retreatment, and the difference in VA change between these groups was not statistically significant (*p* = 0.173). Four eyes had ≥ 15 letters vision loss within year 1, with this due to retinal detachment in 3 cases, and worsening lesion exudation in 1 case.

## Discussion

Trans-scleral retinal cryotherapy as a treatment for RHs was associated with a 58% rate of success (partial or complete) within the first year and a 52% rate of complete success. These results suggest a role for cryotherapy in treating RHs, but no analyzed factors were statistically significant predictors of treatment success.

Previous studies have shown that cryotherapy or vitreoretinal surgery is often used in cases with pre-existing complications, such as exudation or traction [10]. Similarly, large or cluster lesions have been previously thought to be risk factors for worse outcomes/treatment failure, potentially due to their more complex anatomy and the difficulty in fully treating such lesions [10]. Our study demonstrated a trend that single lesions and lesions associated with no or mild SRF at baseline were more likely to achieve treatment success, although these findings did not reach statistical significance in our small cohort. Almost 70% (19/28) of single lesions achieved treatment success, compared to only 13% (1/8) of cluster lesions. Similarly, 70% (19/27) of lesions with no or mild subretinal fluid achieved treatment success, compared to only 11% (1/9) of lesions with increased SRF.

There were a number of cases of retinal detachment, with the majority of these occurring within the first 1 year after cryotherapy. A large number of these involved some degree of tractional detachment, and in these instances, vitrectomy was preferred due to the ability to also treat the tractional component at the time of vitrectomy, as well as the ability to more effectively tamponade the retina.

Although the difference in vision between lesions with and without treatment success was not statistically significant, the magnitude of difference (17.6 letters) is large, and the lack of statistical significance may relate in part to the small sample size. In real world settings, a change of 17–18 letters is likely to be clinically meaningful and represent symptomatic vision changes. This magnitude of change suggests that there may be visual benefits as well as anatomical benefits to achieving treatment success when managing RHs.

Current alternative treatment options for these complex RHs, such as large or cluster lesions, remain limited. Recent series have shown that laser photocoagulation is less likely to be successful in these cases, and surgical excision of RHs has been previously shown to be associated with a high rate of retreatment and reoperation and limited long-term VA [4, 7, 8, 14]. Comparison of treatment modalities has suggested that cryotherapy may have a relatively low success rate, but that PDT and surgical interventions also have high post-operative complication rates [10]. PDT has recently been reported to show considerable success in previously treated lesions and may be an alternate option for some of these RHs, although the majority of these were located in the juxtapapillary or macula region [15]. In the case of PDT, epiretinal membrane formation or transient macula oedema constitutes the majority of reported post-operative complications [10, 15]. Recent meta-analysis has suggested that cryotherapy has a treatment-related complication rate of 27%, vitreoretinal surgery has a complication rate of 57%, PDT a complication rate of 79% and anti-VEGF therapy a complication rate of 100%, although there are particularly low numbers of PDT and anti-VEGF cases [10].These rates of complications can make deciding between interventions difficult, and suggest that cryotherapy may be appropriate for cases which do not yet require vitreoretinal surgery but are not amendable to laser therapy for definitive management. There may also be a degree of selection bias in relation to comparing complication rates, given that some therapies such as PDT or vitrectomy have traditionally been used only in certain subsets of RHs.

Possible new avenues in patients with VHL-related RHs include the use of oral belzutifan, a recently approved systemic hypoxia-inducible factor-2α inhibitor, that was associated with improved retinal lesion appearance, as well as improvement in renal clear cell carcinoma characteristics [16]. Belzutifan may allow for shrinkage and/or stabilization of these more complex lesions, potentially allowing for the use of laser or other therapies, or for more successful cryotherapy to be performed. Given the range of possible treatment options, cryotherapy may benefit from being considered as part of a multitherapeutic approach, particularly given the recent availability of belzutifan, as well as newer anti-VEGF agents with potentially greater efficacy, such as faricimab or high-dose aflibercept. In this setting, cryotherapy may benefit from being considered within a combination of modalities such as belzutifan, anti-VEGF, laser and/or PDT are used to shrink and stabilize lesions prior to administering cryotherapy. However, further research and experience with belzutifan is needed to better determine its role in treating RHs and its place in any such multi-modality treatment algorithm.

There are certain potential advantages of cryotherapy over other currently available treatment modalities, particularly for lesions not amenable to successful treatment with laser. Cryotherapy can often be performed in an office setting and without general anesthesia, which reduces recovery time and limits systemic risk of complications. Additionally, it offers the possibility of a single session treatment for larger lesions, whereas lesions > 1 DD are more likely to need multiple sessions of therapy with laser photocoagulation [4] or other office-based modalities [5, 6]. This advantage is of particular importance in patients for whom travel or attendance at appointments is difficult. Cryotherapy is also anatomically favorable for particularly anterior lesions, where adequate laser or PDT coverage may be difficult. For RHs where these factors may be of increased importance, or where other less invasive treatment modalities may have already proven unsuccessful, cryotherapy remains an important and viable treatment option.

There are limitations to this study. The retrospective nature of the investigation prevented review of standardized timepoints and imaging modalities, and the small sample size with no control group limits the evaluation of outcomes. We aimed to assess the role of cryotherapy in the real-world setting, thus, previously-treated lesions were included in the analysis. Success in these lesions could be attributed to the combination of treatments, rather than cryotherapy alone. Additionally, the inclusion of patients previously treated with cryotherapy may limit the accuracy of the analysis of the treatments analyzed in this series. The majority of lesions occurred in patients with VHL, a condition which predisposed participants to development of multiple lesions and recurrence of lesions that could nonetheless have cumulative consequences such as exudative retinal detachment. We chose to assess success, therefore, at the lesion level where cryotherapy was targeted, but recognize that long term outcomes at the eye level are dependent on multiple factors. Similarly, our primary definition of success allowed for both complete and partial success in order to capture the value of cryotherapy if it resulted in the ability to control the lesion before providing definitive treatment.

This study represents the largest treatment series investigating cryotherapy in RHs published to date and suggests that cryotherapy is effective at controlling some RHs in the short term. Further research is warranted into the optimal usage of cryotherapy as a therapy for RHs, particularly in the setting of newer treatments and the possibility of combination treatment to improve success.

## Figures and Tables

**Table 1:** Initial Demographic, Clinical and Treatment Data (N (%) unless otherwise specified)

Characteristic	Total Cohort	Patients, eyes or lesions without treatment success	Patients, eyes or lesions experiencing complete treatment success	Patients, eyes or lesions experiencing partial treatment success
Patient Characteristics				
Number of patients	24	10	13	1
Female	8 (33.3)	5 (50.0)	3 (23.1)	0 (0.0)
Positive VHL status	21 (87.5)	8 (80.0)	12 (92.3)	1 (100.0)
Age at treatment in years (mean ± SD)	33.8 ± 12.1	28.7 ± 10.2	37.0 ± 12.7	28.0
Average length of follow-up (years)	5.8 ± 3.5	4.2 ± 3.5	6.6 ± 3.2	10.9
Eye and Treatment Characteristics				
Number of eyes	26	11	14	1
Right eye	10 (38.5)	7 (63.6)	3 (21.4)	0 (0.0)
Transconjunctival cryotherapy	17 (65.4)	9 (81.8)	7 (50.0)	1 (100.0)
Operating Room location for cryotherapy	17 (65.4)	9 (81.8)	7 (50.0)	1 (100.0)
Post-operative corticosteroid therapy				
Intravenous	18 (69.2)	9 (81.8)	8 (57.1)	1 (100.0)
Oral	8 (30.8)	2 (18.2)	6 (42.9)	0 (0.0)
Previous cryotherapy treatment to same eye (different RHs)	6 (23.1)	3 (27.3)	2 (14.3)	1 (100.)
Previous laser treatment to same eye	16 (61.5)	6 (54.5)	9 (64.3)	1 (100.)
Previous photodynamic therapy treatment to same eye	2 (7.7)	2 (18.2)	0 (0.0)	0 (0.0)
Previous anti-vascular endothelial growth factor treatment to same eye	6 (23.1)	4 (36.4)	2 (13.3)	0 (0.)
Previous pars plana vitrectomy to same eye	4 (15.4)	2 (18.2)	2 (13.3)	0 (0.0)
Previous scleral buckle to same eye	5 (19.2)	2 (18.2)	2 (14.3)	1 (100.0)
Visual acuity (Mean ETDRS letters ± SD)	69.7 ± 20.4 [20/40]	59.4 ± 27.2 [20/63]	77.0 ± 8.7 [20/25]	80.0
≥ 20/40	19 (73.1)	5 (45.5)	13 (92.9)	1 (100.0)
≥ 20/80, < 20/40	1 (3.8)	1 (9.1)	0 (0)	0 (0.0)
≥20/200, < 20/80	4 (15.4)	3 (27.3)	1 (7.1)	0 (0.0)
<20/200	2 (7.7)	2 (18.2)	0 (0.0)	0 (0.0)
Baseline Lesion Characteristics				
Number of treated lesions	36	16	17	3
Follow-up period for lesions (Mean years ± SD)*	6.7 ± 3.5	5.5 ± 3.9	6.6 ± 3.2	10.9
RH lesion type				
Single	28 (77.8)	9 (56.3)	16 (94.1)	3 (100.0)
Cluster	8 (22.2)	7 (43.8)	1 (5.9)	0 (0.0)
Size of RH (disc diameters)				
1	6 (16.7)	3 (18.8)	2 (11.8)	1 (33.3)
2	20 (55.6)	9 (56.3)	10 (58.8)	1 (33.3)
> 3	10 (27.8)	4 (25.0)	5 (29.4)	1 (33.3)
Lipid exudation				
None	13 (36.1)	5 (31.3)	8 (47.1)	0 (0.0
Mild	8 (22.2)	1 (6.3)	6 (35.3)	1 (33.3)
Moderate or Severe	15 (41.7)	10 (62.5)	3 (17.6)	2(66.7)
Vascular dilation				
None,	1 (2.8)	0 (0.0)	1 (59.9)	0 (0.0)
Mild	2 (5.6)	1 (6.3)	1 (5.9)	0 (0.0)
Moderate	7 (19.4)	3 (18.8)	3 (17.6)	1 (33.3)
Severe	26 (72.2)	12 (75.0)	12 (70.6)	2 (67.7)
Subretinal fluid				
None	14 (38.9)	2 (12.5)	11 (64.7.0)	1 (33.3)
Mild	13 (36.1)	6 (37.5)	6 (35.3)	1 (33.3)
Moderate	3 (8.3)	2 (12.5)	0 (0.0)	1 (33.3)
Severe	2 (5.6)	2 (12.5)	0 (0.0)	0 (0.0)
Exudative Detachment	4 (11.1)	4 (25.0)	0 (0.0)	0 (0.0)
Location (Quadrant)				
Superotemporal	19 (52.8)	8 (50.0)	10 (58.8)	1 (33.3)
Inferotemporal	10 (27.8)	4 (25.0)	4 (23.5)	2 (66.7)
Superonasal	4 (11.1)	4 (18.9)	1 (5.9)	0 (0.0)
Inferonasal	3 (8.3)	1 (6.3)	2 (11.8)	0 (0.0)
Location (Position)				
Posterior to equator	8 (22.2)	4 (25.0)	3 (17.6.)	1 (33.3)
Equatorial	20 (55.6)	10 (62.5)	9 (52.9)	1 (33.3)
Anterior to equator	8 (22.2)	2 (12.5)	5 (29.4)	1 (33.3)
Traction present	7 (19.4)	4 (25.0)	2 (11.8)	1 (33.3)

*N* = 33 eyes* (3 lesions were present and treated at baseline but did not have ongoing follow up data)

ETDRS: Early Treatment of Diabetic Retinopathy Study

RH: Retinal Hemangioblastoma

VHL: Von-Hippel Lindau

**Table 2:** Clinical Outcomes and Change in Functional and Lesional Parameters By 1 Year

	Eyes with VA available at 1 year	Eyes without treatment success of all treated lesions	Eyes with complete treatment success of all treated lesions	Eyes with partial treatment success of all treated lesions
Ocular Characteristic				
Number of eyes	21	9	9	3
Change in visual acuity in Letters (mean ± SD)	-4.6 ± 14.5	-3.0 ± 13.2	-0.6 ± 7.5	-21.3 ± 26.1
Change in vision**				
Improved (5 letters or more gain)	5 (23.8)	3 (33.3)	2 (22.2)	0 (0.0)
Stable (± 5 letters)	10 (47.6)	4 (44.4)	5 (55.6)	1 (33.3)
Reduced (−5 to −15 letters)	6 (28.6)	2 (22.2)	2 (22.2)	0 (0.0)
Significantly Reduced (≥ 15 letters lost)	4 (19.0)	2 (22.2)	0 (0.0)	2 (66.7)
Visual acuity level maintained				
Vision Maintained > 20/40	12 (57.1)	3 (33.3)	8 (88.9)	2 (66.7)
Vision Maintained > 20/200 and < 20/40	3 (14.3)	2 (22.2)	1 (11.1)	1 (33.3)
New retinal detachment	5 (23.8)	3 (33.3)	0 (0.0)	2 (16.7)
		Lesions not experiencing treatment success	Lesions experiencing treatment success	
Lesion Characteristic*				
Change in lipid exudation				
N	21 lesions of 19 eyes	10	8	3
Reduced	8 (38.1)	4 (40.0)	2 (25.0)	2 (66.7)
Stable	11 (52.4)	4 (40.0)	6 (75.0)	1 (33.3)
Increased	2 (9.5)	2 (20.0)	0 (0)	0 (0.0)
Change in subretinal fluid				
N	25 lesions of 21 eyes	10	12	3
Reduced	9 (36.0)	6 (60.0)	2 (16.7)	1 (33.3)
Stable	12 (48.0)	2 (20.0)	9 (75.0)	1 (33.3)
Increased	4 (16.0)	2 (20.0)	1 (8.3)	1 (33.3)
Change in vascular dilation				
N	19 lesions of 17 eyes	9	7	3
Reduced	10 (52.6)	4 (44.4)	4 (57.1)	2 (66.7)
Stable	8 (42.1)	4 (44.4)	3 (42.9)	1 (33.3)
Increased	1 (5.3)	1 (11.1)	0 (0)	0 (0.0)

* Different N values due to missing data and inability to assess for certain features in certain eyes

** Due to overlapping characteristic groups, N values and % may differ

VA: Visual Acuity

SD: Standard Deviation

**Table 3:** Age-adjusted Proportional Hazards Regression for Baseline Factors Investigated for Possible Association with Treatment Success^*^

	Univariate** (Hazard Ratio and 95% Confidence Interval)	*P* *Value*
Age (per 1 year increase)	1.085 (0.998, 1.180)	0.0032
Male gender (vs. female)	7.80 (0.84, 72.71)	0.0712
Cluster lesion type (vs. single)	0.15 (0.01, 1.64)	0.1217
Lesion location (vs. composite of superonasal, inferonasal, and temporal locations***)		
Inferotemporal	2.49 (0.27, 22.84)	0.4211
Superotemporal	2.15 (0.390, 15.412)	0.4478
Lesion size (vs. <1DD)		
(1–2DD)	3.30 (0.40, 27.54)	0.2700
≥ 3DD)	1.14 (0.11, 11.56)	0.9115
Severe feeding/draining vessel dilation (vs. mild or moderate***)	1.39 (0.26, 7.37)	0.6987
Subretinal fluid presence (vs. absence)	0.10 (0.01, 1.03)	0.0527
Lipid exudate (vs. none)		
Mild or Moderate***	4.93 (0.42, 57.99)	0.2048
Severe	0.52 (0.09, 3.05)	0.4703
Vitreous traction presence (vs. absence)	0.32 (0.05, 2.03)	0.2269
Lesion fibrosis (vs. none)		
Mild or Moderate	1.46 (0.24, 8.89)	0.6786
Severe	0.52 (0.07, 3.95)	0.5288
Intravenous corticosteroid (vs. oral)	0.77 (0.14, 4.22)	0.7608
Conjunctival peritomy performed (vs. not performed)	0.67 (0.12, 3.64)	0.6391
Operating room procedure (vs. clinic)	0.67 (0.12, 3.64)	0.6391
Previous cryotherapy (vs. no previous cryotherapy)	0.31 (0.05, 2.10)	0.2295

* Lesions with partial or complete success were combined (17 complete success and 3 partial success)

** Age-adjusted with the exception of the Age variable

*** Grouped for analysis purposes

DD: Disc Diameter

## Data Availability

Available from the authors upon reasonable request.

## Key messages

### What is known

- Hemangioblastomas can be treated with ablative cryotherapy but success rates and factors affecting these are not known.

### What is new

- This paper analyzes success rates and shows a success rate of 57.4% of retinal cryotherapy, with stabilization of vision. Apart from lower patient age at the time of therapy, no other patient or lesions factors were suggestive of treatment success.
